# Characterisation of δ-Conotoxin TxVIA as a Mammalian T-Type Calcium Channel Modulator

**DOI:** 10.3390/md18070343

**Published:** 2020-06-30

**Authors:** Dan Wang, S.W.A. Himaya, Jean Giacomotto, Md. Mahadhi Hasan, Fernanda C. Cardoso, Lotten Ragnarsson, Richard J. Lewis

**Affiliations:** 1 Institute for Molecular Bioscience, The University of Queensland, Brisbane, QLD 4072, Australia; dan.wang@imb.uq.edu.au (D.W.); h.siddhihalu@imb.uq.edu.au (S.W.A.H.); mahadhi.hasan@imb.uq.edu.au (M.M.H.); f.caldascardoso@imb.uq.edu.au (F.C.C.); l.ragnarsson@imb.uq.edu.au (L.R.); 2 Queensland Brain Institute, The University of Queensland, Brisbane, QLD 4072, Australia; j.giacomotto@uq.edu.au; 3 Queensland Centre for Mental Health Research, West Moreton Hospital and Health Service and University of Queensland, Brisbane, QLD 4072, Australia

**Keywords:** TxVIA, mammalian Na_V_ channel, selective inhibitor, T-type Ca_V_3.2

## Abstract

The 27-amino acid (aa)-long δ-conotoxin TxVIA, originally isolated from the mollusc-hunting cone snail *Conus textile*, slows voltage-gated sodium (Na_V_) channel inactivation in molluscan neurons, but its mammalian ion channel targets remain undetermined. In this study, we confirmed that TxVIA was inactive on mammalian Na_V_1.2 and Na_V_1.7 even at high concentrations (10 µM). Given the fact that invertebrate Na_V_ channel and T-type calcium channels (Ca_V_3.x) are evolutionarily related, we examined the possibility that TxVIA may act on Ca_V_3.x. Electrophysiological characterisation of the native TxVIA on Ca_V_3.1, 3.2 and 3.3 revealed that TxVIA preferentially inhibits Ca_V_3.2 current (IC_50_ = 0.24 μM) and enhances Ca_V_3.1 current at higher concentrations. In fish bioassays TxVIA showed little effect on zebrafish behaviours when injected intramuscular at 250 ng/100 mg fish. The binding sites for TxVIA at Na_V_1.7 and Ca_V_3.1 revealed that their channel binding sites contained a common epitope.

## 1. Introduction

The δ-conotoxin TxVIA (King Kong peptide), a 27-amino acid (aa)-long peptide with six cysteine residues, was originally isolated from the mollusc-hunting cone snail species *Conus textile* [1]. The unique name of TxVIA, “King Kong peptide”, stems from the dominant posture lobsters adopt following injection of the toxin, although it has also been observed to produce convulsive-like activity in snails [1]. TxVIA also produced a paralytic effect in molluscs (*Patella*) but not in fish (*Cambusia*), insects (*Sarcophaga*) or crustaceans (*Porcellio*) [2]. Although no mammalian activity has been reported, TxVIA was shown to potently slow Na_V_ channel inactivation in molluscs [3]. Previous binding and electrophysiological studies suggest TxVIA binds to both mollusc and rat brain Na_V_s at sites adjacent to the binding sites of conotoxin CsTx and coral toxin GPT [4,5,6]. However, TxVIA binding to mammalian Na_V_ channels is non-functional [4]. The disparate sequence alignment of the *Aplysia* Na_V_ channel with hNa_V_1.7 (44% similarity overall, with especially poor overlap across the extracellular regions) supports the distinct modes of action at these distantly related sodium channels.

T-type calcium channels (Ca_V_3.x) have been identified with two ion-selectivity filters [7], which allow permeability to Na^+^ ions in the absence of Ca^2+^ ions, with genomic studies on the jellyfish Na_V_ channel, revealing an ancestral resemblance to Ca_V_3.x [8]. Ca_V_3.x provides a privileged gate for calcium influx that initiates many physiological events including secretion, neurotransmission and cell proliferation [9,10], implicating it in many pathophysiological disorders and diseases, including absence epilepsy, Parkinson’s disease (PD), hypertension, cardiovascular diseases, cancers and pain [11]. The evolutionary relationship between the invertebrate Na_V_ channel with Ca_V_3.x raised the possibility that TxVIA may modulate Ca_V_3.x.

In this work, we identified the spatial distribution of TxVIA in the *C. textile* venom duct, isolated and characterised native TxVIA at human Ca_V_3.x using Fluorescent Imaging Plate Reader (FLIPR) and electrophysiological (QPatch) assays, confirmed the lack of activity of TxVIA on human Na_V_ channels endogenously expressed in SH-SY5Y cells [12] and mouse Na_V_1.7, and used zebrafish [13,14] to analyse behavioural effects using an automated tracking device (i.e. Zebrabox). Finally, we compared the binding sites for TxVIA predicted from molecular docking studies using homology models of Na_V_1.7 and Ca_V_3.1.

## 2. Results

### 2.1. Distribution, Isolation and Identification of Native TxVIA

**Figure 1:**
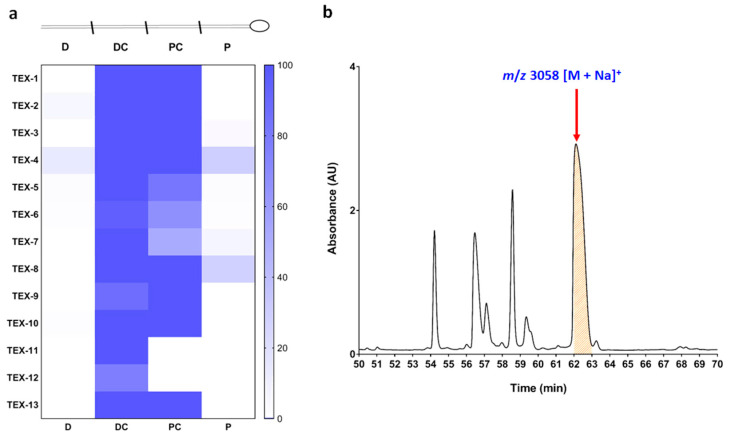
(**a**) TxVIA distribution across the four venom duct sections (distal (D), distal central (DC), proximal central (PC), proximal (P)) of 13 *Conus textile* specimens. (**b**) Partial chromatogram of TEX-4 DC section fractionation. The *x*-axis represents the retention time, the *y*-axis represents UV absorption at 214 nm, and the red arrow indicates TxVIA eluting at 62.5 min (mass data obtained by MALDI-TOF MS).

*C. textile* venom ducts of thirteen specimens (TEX-1–13) were dissected into distal (D), distal central (DC), proximal central (PC) and proximal (P) sections, and the extracted venom from each section was analysed by liquid chromatography/mass spectrometry (LC/MS). TxVIA expression across the thirteen specimens (Figure 1a) was localised to the central portions of the *C. textile* venom duct. Guided by TxVIA distribution, the distal central venom of TEX-4 was selected for fractionation (Figure 1b). Native TxVIA was isolated and its amino acid sequence WCKQSGEMCNLLDQNCCDGYCIVLVCT confirmed by tandem mass spectrometry (MS/MS) analysis.

### 2.2. Evaluation of Mammalian Na_V_ Channel Activitiy of TxVIA using FLIPR Cell-Based Assays

**Figure 2:**
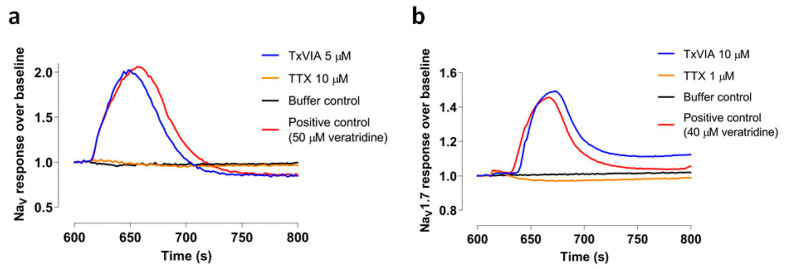
Characterisation of TxVIA in sodium channels. (**a**) Representative fluorescent traces of the hNa_V_ responses with and without the addition of 5 µM TxVIA. (**b**) Representative fluorescent traces of the mouse Na_V_1.7 responses with and without the addition of 10 µM TxVIA.

TxVIA (5 µM) was tested in SH-SY5Y cells using a FLIPR assay to measure endogenously expressed Na_V_1.2 and Na_V_1.7 [12]. Although 0.5 µM TxVIA has previously been shown to slow inactivation of molluscan Na_V_ current [3], 5 µM TxVIA did not produce any detectable effect on human Na_V_ responses in SH-SY5Y cells (*n* = 3, *p* = 0.37) (Figure 2a). TxVIA (10 µM) also failed to significantly modify calcium influx in HEK cells transiently expressing mouse Na_V_1.7 (*n* = 2, *p* = 0.29) (Figure 2b).

### 2.3. Pharmacological Characterisation of TxVIA in Ca_V_3.x

**Figure 3:**
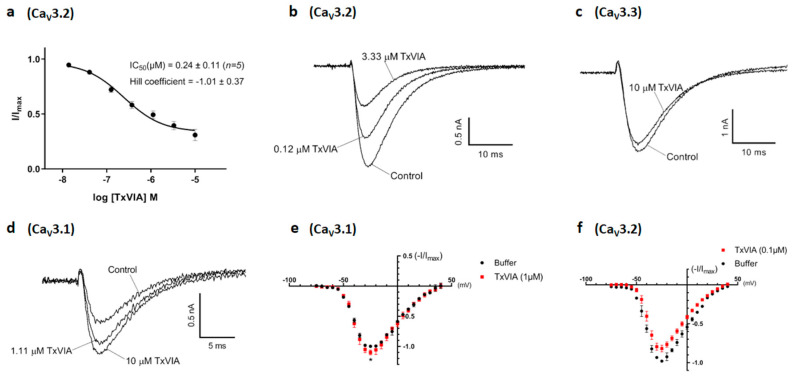
Modulation of Ca_V_3.1, Ca_V_3.2 and Ca_V_3.3 current by TxVIA. (**a**) Concentration response curves of TxVIA on recombinant hCa_V_3.2 channels (*n* = 5) using the QPatch. Data are means ± SEM. (**b**) Representative Ca_V_3.2 I_Ca_ during 200 ms depolarisations to V_max_ (−20 mV) from a holding potential of −90 mV before and after perfusions of 0.12 µM and 3.33 µM of TxVIA, as indicated. (**c**) Representative Ca_V_3.3 I_Ca_ during 200 ms depolarisations to V_max_ (−10 mV) from a holding potential of −90 mV before and after perfusions of 10 µM of TxVIA, as indicated. (**d**) Representative Ca_V_3.1 I_Ca_ during 200 ms depolarisations to V_max_ (−20 mV) from a holding potential of −90 mV before and after perfusions of 1.11 µM and 10 µM of TxVIA, as indicated. (**e**) Normalised I–V relationships of Ca_V_3.1 (*n* = 5) plotted from –75 mV to +40 mV before (black) and after (red) the addition of 1 µM of TxVIA, V_max_ at −25 mV (10.0% ± 3.9% current enhancement, *p* = 0.04), *I_max_* at 0.12 ± 0.01 nA. Data are means ± SEM. Single-voltage protocol was applied in between to measure the TxVIA effect. (**f**) Normalised I–V relationships of Ca_V_3.2 (*n* = 4) plotted from −75 mV to +40 mV before (black) and after (red) the addition of 0.1 µM of TxVIA, V_max_ at −25 mV, I_max_ at 0.65 ± 0.04 nA. Data are means ± SEM. Single-voltage protocol was applied in between to measure the TxVIA effect.

We examined the effects of native TxVIA on human Ca_V_3.x by whole-cell patch-clamp using the automated electrophysiology platform QPatch 16 X (Figure 3). Whereas TxVIA partially inhibited Ca_V_3.2 (*n* = 5) (Figure 3a,b) at high nanomolar concentrations, it had little effect on Ca_V_3.3 (*n* = 6) (Figure 3c) and promoted the opening of Ca_V_3.1 (*n* = 5) (Figure 3d). Current-voltage (*I–V*) relationships of Ca_V_3.1 in the presence of 1 µM TxVIA revealed that the channel modulation was not accompanied by shifts in the *I–V* relationship (*n* = 5, *p* = 0.63) (Figure 3e). Similarly, 0.1 µM TxVIA did not shift the *I–V* relationship of Ca_V_3.2 (*n* = 4, *p* = 0.21) (Figure 3f). We also tested native TxVIA in the Ca_V_3.2 FLIPR window current assay [15], where 60 µM TxVIA only showed partial (42%) inhibition (*n* = 3) (data not shown).

### 2.4. TxVIA Docking in Human Na_V_1.7 and Ca_V_3.x

**Figure 4:**
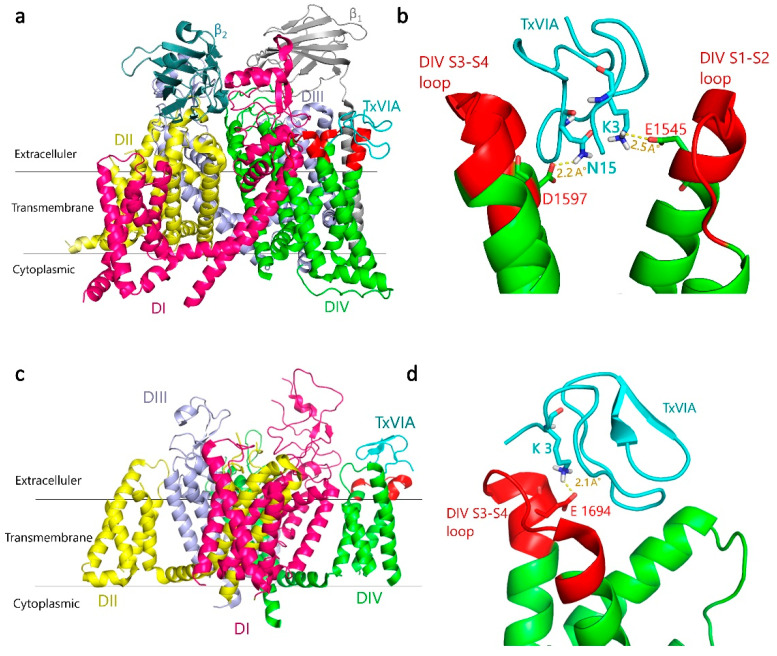
Predicted binding mode of TxVIA (coloured cyan) in human Na_V_1.7 and Ca_V_3.1. (**a**) General view of the lowest energy docking pose of TxVIA binding to hNa_V_1.7 DIV S3-S4 and S1-S2 linkers (extracellular loops, coloured red). (**b**) Local view of TxVIA interactions highlighting that D1597 in hNa_V_1.7 DIV S3-S4 linker and E1545 in DIV S1-S2 linker make close contact with N15 and K3 of TxVIA, respectively. (**c**) General view of the lowest energy docking pose of TxVIA binding to hCa_V_3.1 DIV S3-S4 linker (coloured red). (**d**) Local view of the close interaction between K3 in TxVIA and E1694 in the hCa_V_3.1 DIV S3-S4 linker. Predicted hydrogen bonds are shown as yellow dashed lines, with distances shown in Å.

Previous binding and electrophysiological studies have suggested that TxVIA binds to a variety of Na_V_s despite its lack of functional effects on rat brain Na_V_s [4], and human Na_V_1.2 and Na_V_1.7 responses (present study). The binding site of TxVIA is expected to be extracellular, likely adjacent to but distinct from neurotoxin site 3 [4,5,6]. Site 3 was initially recognised to be located in the S5-S6 linker of domain I (DI) and IV (DIV) [16], and a later study identified that additional residues in the DIV S3-S4 linker influenced α-scorpion and sea anemone toxin binding [17,18]. Based on this background, we docked TxVIA to the DIV S3-S4 linker of the cryo-electron microscopy (Cryo-EM) structure of human Na_V_1.7-β1-β2 complex (Protein Data Bank (PDB) code 6J8I) [19]. This docking generated a docking pose where TxVIA fits between the DIV S1-S2 and S3-S4 linkers of Na_V_1.7 (Figure 4a,b) (Table 1). Importantly, this binding pose identified a strong salt bridge between K3 in TxVIA and E1545 in the DIV S1-S2 linker that is a likely key binding determinant (Figure 4b).

The 30-aa spider toxin ProTx-II, initially identified as a Na_V_1.7 selective blocker [20], has also been shown to selectively block the hCa_V_3.2 current among the three Ca_V_3.x subtypes [21]. As a well-established site 4 (DII S3-S4 linker) toxin [22], the crystal structure of the ProTx-II-DII hNa_V_1.7 complex [23] has been generated. It has been suggested that TxVIA binds to a different binding site from ProTx-II [4], which was also supported by our docking results with unfavourable binding affinity (Table 1).

We also explored TxVIA binding in hCa_V_3.x. The MolProbity score of Ca_V_3.2 and Ca_V_3.3 homology models generated from the recently reported hCa_V_3.1 Cryo-EM structure [24] were 1.97 and 1.98 respectively, indicating the high quality of the modelled structures. An assessment of the Ramachandran plot showed that only 1.85% and 1.34% residues fell in outlier regions for Ca_V_3.2 and Ca_V_3.3 models, respectively, with no outliers found for residues in the DIV S3-S4 linker region.

The selective inhibition of ProTx-I [21] for Ca_V_3.1 current over Ca_V_3.2 has been identified to be partly attributed to the DIV S3-S4 linker in T-type Ca_V_s [25]. Interestingly, our docking results also suggest that TxVIA would bind to the DIV S3-S4 linker of Ca_V_3.1 (Figure 4c,d) with higher affinity than to Ca_V_3.2 and Ca_V_3.3 (Table 1). However, the possibility that the DIV S3-S4 linker may contribute to the activation of Ca_V_3.1 by TxVIA requires further study. Similar to its docking at Na_V_1.7, the best docking pose for TxVIA binding to Ca_V_3.1 shows TxVIA interacting between DIV S1-S2 and S3-S4 linkers of Ca_V_3.1. As shown in Figure 4d, a strong salt bridge was again identified between K3 in TxVIA and E1694 in the Ca_V_3.1 DIV S3-S4 linker, although no interactions were identified between TxVIA and the S1-S2 in Ca_V_3.1 DIV.

### 2.5. Behavioural Analysis on Zebrafish after Intramuscular Injection of TxVIA

Although the TxVIA inhibition of Ca_V_3.2 indicated possible pain-relieving activity, its activation of Ca_V_3.1 indicated a possible pain inducing activity, albeit at higher concentrations. We also showed that TxVIA had no effect on the human pain related Na_V_1.7. Given the potential of TxVIA to play a defensive role, we examined its effect on zebrafish at concentrations up to 250 ng/100 mg fish. However, TxVIA could have different pharmacology on zebrafish and mammalian Ca_V_s and Na_V_s that may influence interpretation of responses in zebrafish.

**Figure 5:**
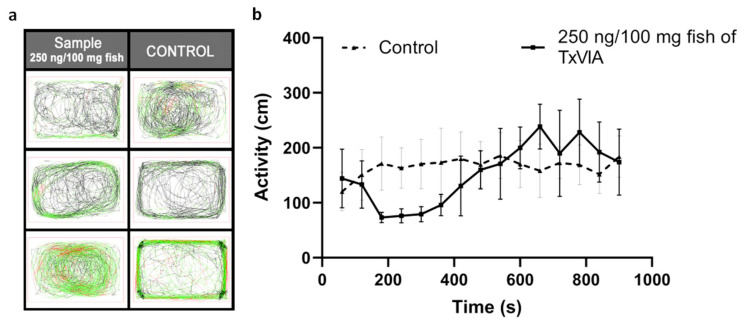
TxVIA-induced behavioural response in adult zebrafish (*n* = 3). (**a**) The six boxes illustrate the 15 min swimming tracks of the control zebrafish (*n* = 3) and the zebrafish injected with 250 ng/100 mg fish of TxVIA (*n* = 3), respectively. The fish generally start with a comparatively slow swimming speed, indicated in black lines, and end with a regular swimming speed, indicated in green lines. Erratic or fast swimming tracks are indicated in red lines. (**b**) The adult zebrafish injected with 250 ng/100 mg fish of TxVIA (continuous line) showed a reduced activity (measured by distance travelled per min) in the first 8 min compared to the activity of the control fish injected with saline sterilised water (dotted line), followed by a small burst of activities for 5 min, and returned to normal gradually. Data are means ± SEM.

None of the injected fish showed signs indicative of pain-related behaviours or paralysis after the injection (*n* = 3, *p* = 0.37), and no adverse effects were observed in the 24 h post-injection. Fish swimming tracks were also recorded during the first 15 min post-injection (see Figure 5). Zebrafish injected with 250 ng/100 mg fish of TxVIA showed reduced swimming activity compared to control fish in the first 8 min, however this effect did not reach significance and soon reversed to normal edge swimming. Additionally, the absence of swimming bursts or erratic swimming behaviour after TxVIA injection indicates that pain pathways were not activated.

## 3. Discussion

Conotoxins are potent and selective modulators of mammalian ion channels and receptors. The King Kong peptide TxVIA has previously been characterised as a mollusc Na_V_ channel modulator, producing convulsive-like activity in snails [1] and paralytic effects in the mollusc *Patella* sp. [2]. In this study, we characterised the effects of TxVIA on human Na_V_s and Ca_V_3.x, and zebrafish behaviours. These studies revealed for the first time that TxVIA is a nM inhibitor of Ca_V_3.2 with activating activity on Ca_V_3.1 at µM concentrations. Interestingly, high concentrations of TxVIA (5 µM) were inactive on endogenously expressed Na_V_s in SH-SY5Y cells, including Na_V_1.2 and Na_V_1.7, or heterologously expressed mouse Na_V_1.7 (10 µM). In addition, our experiments found that TxVIA does not trigger pain-like behaviours or paralysis in zebrafish at up to 250 ng/100 mg fish, consistent with the lack of an excitatory effect on vertebrate Na_V_s. Although TxVIA did not target the Ca_V_3.x window current like Ca_V_3.x small molecule blockers [26,27], electrophysiological characterisation of TxVIA revealed it to be a nM inhibitor for Ca_V_3.2 (IC_50_ = 0.24 µM). Ca_V_3.2 is considered to be a promising novel therapeutic target for pain with Ca_V_3.2 playing a major pronociceptive role in spinal nociceptive neurons and primary afferents [28,29,30].

Our LC-MS analysis of dissected *C. textile* venoms revealed that TxVIA was expressed at its highest levels in the central region of the venom duct as the dominant component. Previous studies from our laboratory have revealed that *Conus geographus* and *Conus marmoreus* have evolved to produce defensive and predatory venoms from the proximal and distal regions of the venom duct, respectively, that are deployed in response to the corresponding stimuli [31]. However, the role of central regions of the venom duct in these processes remains unknown. In future studies, we aim to investigate the contribution of TxVIA to the defensive and predatory milked venoms of *C. textile* to unravel its role in defensive and/or predatory behaviour.

To gain insight into TxVIA binding to Na_V_ and Ca_V_ channels, we performed molecular docking studies of TxVIA binding to Cryo-EM structures of hNa_V_1.7 and hCa_V_3.1. A strong salt bridge was identified in TxVIA binding to the DIV S1-S2 linker of Na_V_1.7, suggesting that the DIV S1-S2 linker could be a new binding site affecting Na_V_ channel targeting neurotoxins. Our docking studies of TxVIA in hCa_V_3.x showed that TxVIA binds to the DIV S3-S4 linker of Ca_V_3.1 with a strong salt bridge, whereas it failed to dock to the DIV S3-S4 linker of Ca_V_3.2 and Ca_V_3.3. These results suggested that the interaction sites between TxVIA and hCa_V_3.x may differ among the three subtypes, which is supported by our electrophysiological data showing it has differential effects across the three subtypes. We speculate that the DIV S3-S4 linker may contribute to both the selective inhibition [25] and activation of the Ca_V_3.1 current.

**Figure 6:**
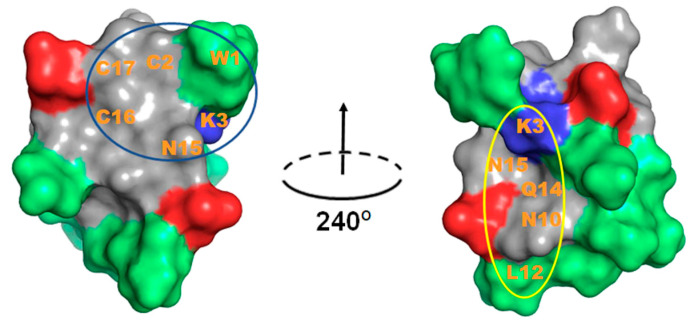
TxVIA (PDB 1FU3) structure pair obtained by 240° horizontal rotation. Peptide surface presented with blue and red colours indicate positive and negative charged residues, respectively, and green colour indicates hydrophobic uncharged residues. The predicted buried surface of TxVIA binding to Na_V_1.7 is circled in blue and the predicted buried surface of TxVIA binding to Ca_V_3.1 is circled in yellow. The predicted interacting residues are labelled.

Toxins with the inhibitor cystine knot (ICK) motif have been mostly recognised as modulators of voltage-gated ion channels [32]. However, ICK peptides with related folds but different sequences often show altered selectivity for ion channels, indicative of promiscuous pharmacophore interactions. δ-Conotoxin TxVIA has been reported to show a prominent hydrophobic patch covering one side of the peptide surface (Figure 6), which was proposed to be crucial for sodium channel binding [33], with our work confirming that TxVIA is non-functional at mammalian Na_V_s. Interestingly, our docking results reveal that the side opposite to the prominent hydrophobic patch of TxVIA is involved in its binding to DIV extracellular loops of Na_V_1.7 (Figure 6), suggesting the hydrophobic patch in TxVIA only contributes to Na_V_ channel affinity. Other Ca_V_3.x peptide blockers with selectivity for Ca_V_3.2 include two hydrophobic tarantula toxins PsPTx3 [34,35] and ProTx-II [21] which also inhibits Na_V_1.7 [20]. Previous structure activity relationship (SAR) studies on ProTx-II reveal that hydrophobic patch interactions with membrane lipids are required for high affinity interactions with hNa_V_1.7 [36] and Na_V_1.5 [37]. Ca_V_3.2 selective peptide blockers also typically show a prominent hydrophobic face, whereas non-hydrophobic peptide blocker ProTx-I inhibits Ca_V_3.1 [21,25,38]. Indeed, our docking studies suggest that TxVIA also binds to the DIV S3-S4 linker of Ca_V_3.1 through a more polar surface and not through the adjacent prominent hydrophobic patch (Figure 6).

In conclusion, we have identified that δ-conotoxin TxVIA modulates mammalian Ca_V_3.x, but not mammalian Na_V_ channels. TxVIA represents a promising new tool to improve our understanding of the molecular mechanism and determinants of activation and inactivation of the different Na_V_1.x and Ca_V_3.x subtypes.

## 4. Materials and Methods 

All reagents were used as purchased from Sigma-Aldrich without further purification.

### 4.1. LC/MS Analysis of TxVIA Distribution in the C. textile Venom Duct

Thirteen adult *C. textile* specimens collected from One Tree Island on the Great Barrier Reef (Queensland, Australia) were sacrificed and dissected into four sections on ice. The crude venom was extracted into 30% acetonitrile, acidified with 0.1 formic acid. The collected 52 crude *C. textile* samples from each of the four duct sections (5 µL) were chromatographically separated on an ultra HPLC system (Shimadzu Scientific, Rydalmere, Australia) directly coupled to a 5600 TripleTOF MS (SCIEX, Foster City, USA). The LC separation was achieved using a Zorbax C_18_ 4.6 × 150 mm column at a linear 1.3% B (acetonitrile/0.1% formic acid (aq)) min^−1^ gradient with a flow rate of 0.2 ml min^−1^ over 90 min. Data were acquired over a time-of-flight (TOF) mass range of 350–2200 Da with an ion spray voltage of 5500 V (CUR 25, TEM 500, GS1 50 and GS2 60). Acquired data were then analysed with Sciex Analyst^TF^ 1.6 software.

### 4.2. C. textile Crude Venom Fractionation for the Collection of Native TxVIA

Solvent A consists of 0.05% trifluoroacetic acid (TFA) in milli-Q water, whereas solvent B consists of 0.043% TFA and 90% acetonitrile in water. 1.4 mg of lyophilised *C. textile* crude venom dissolved in water with 30% solvent B was loaded using an UltiMate 3000 analytical autosampler (Dionex, Sunnyvale, CA) onto a 00G-4053-E0 *Jupiter*® (Phenomenex, Torrance, CA, USA) 5 µm *C18* 300 Å, 250 × 4.6 mm analytical reversed phase high performance liquid chromatography (RP-HPLC) column and eluted at a flow rate of 0.7 mL/min over 100 min. The elution was monitored at 214 nm.

The following gradient generated by an UltiMate 3000 pump was used to fractionate the *C. textile* crude venom: A constant 5% solvent B over 5 min, 5–80% solvent B over 75 min, 80–90% solvent B over 1 min, a constant 90% solvent B over 4 min, 90–5% solvent B over 1 min, and a constant 5% solvent B over 1 min.

A solvent blank run using the same gradient and equilibration with 5% solvent B for 15 min preceded each separation. Fractions were collected every 1 min over 80 min with a Gilson FC 204 automatic fraction collector (Gilson, Middleton, WI). Collected fractions were transferred into 1.5 mL Eppendorf tubes, dried in a speed vacuum concentrator, resuspended in 100 µL of milli-Q water, vortexed and stored at −20 ℃ prior to assaying. Peptide concentrations were measured using the NanoDrop One (Thermo Scientific, MA, US). All solvents used were HPLC grade.

### 4.3. MS and MS/MS Analysis and Sequence Determination of Native TxVIA

#### 4.3.1. MALDI-TOF Mass Spectrometry

Venom peptide masses were verified by matrix-assisted laser desorption/ionisation time-of-flight mass spectrometry (MALDI-TOF MS) using the 4700 Proteomics Bioanalyzer (Applied Biosystems, CA, USA). Venom fractions in water obtained from RP-HPLC were mixed with the matrix CHCA (5 mg/mL in 50% ACN, 1% FA) in 1:1 (v/v) ratio and spotted on a MALDI plate. MALDI-TOF spectra were collected in reflector positive mode and the reported masses are monoisotopic M + H^+^ ions.

#### 4.3.2. Reduction and Alkylation of Cysteine Residues

45 µL peptide solution was reduced and alkylated with 50 µL of reduction/alkylation cocktail (containing 97.5% acetonitrile, 2% iodoethanol, and 0.5% triethylphosphine by volume) in 50 mM ammonium carbonate solution (pH 11), capped and incubated at 37 °C for 2 h. The sample was then uncapped and evaporated on a speed vacuum for more than 1 h. Dried samples were kept in −80 ℃ before use.

#### 4.3.3. Trypsin Digestion

Reduced and alkylated peptide was reconstituted with 20 µL of 50 mM ammonium bicarbonate solution (pH 8). Peptide digestion was achieved in 40 ng/µL modified sequencing grade trypsin (Promega, Madison, WI, USA) incubated at 37 °C overnight. Digestion was terminated by adding 5 µL of 5% formic acid.

#### 4.3.4. LC-MS/MS Analysis and Sequence Determination of Native TxVIA

The tryptic peptides (15 µL) were analysed with chromatographic separation using an ultra HPLC system (Shimadzu Scientific, Rydalmere, Australia) directly coupled to a 5600 TripleTOF MS (SCIEX, Foster City, USA). Data were acquired for 55 min 2 s with an ion spray voltage of 5500 V (CUR 25, TEM 500, GS1 50 and GS2 60). ProteinPilot™ 4.0 software (SCIEX) with the Paragon Algorithm was used for protein identification. Tandem mass spectrometry (MS/MS) data was searched against database of *C. textile* from conoserver (http://conoserver.org/). The search parameters were defined as iodoethanol modified for cysteine alkylation and trypsin as the digestion enzyme.

### 4.4. Cell Culture and Transient Expression

The human embryonic kidney 293 (HEK293) cell line expressing human Ca_V_3.2 or Ca_V_3.3 (kind gift from Emmanuel Bourinet, University of Montpellier, France) were cultured under 5% carbon dioxide at 37 °C in Dulbecco’s modified Eagle’s medium (DMEM), Glutamax (Gibco, Life Technologies, Carlsbad, CA, US) supplemented with 10% (v/v) fetal bovine serum (FBS), 100 U/mL penicillin, 100 μg/mL streptomycin (Gibco, Life Technologies) and 750 μg/mL geneticin (G418) (Gibco, Life Technologies). The Chinese hamster ovary (CHO) cell lines (Emmanuel Bourinet, Montpellier, France) expressing human Ca_V_3.1 were cultured under 5% carbon dioxide at 37 °C in Minimum Essential Medium Eagle-alpha modification (α-MEM) Glutamax (Gibco, Life Technologies), supplemented with 10% (v/v) FBS and 300 μg/mL geneticin (G418) (Gibco, Life Technologies). The human neuroblastoma SH-SY5Y cells (Victor Diaz, Goettingen, Germany) were cultured under 5% carbon dioxide at 37 °C in RPMI 1640 antibiotic-free medium (Invitrogen, Carlsbad, CA, US), supplemented with 15% FBS and 2 mM GlutaMAX™ (Invitrogen). Dulbecco’s phosphate-buffered saline (DPBS) (Gibco, Life Technologies) was used to wash the cells, and 0.25% Trypsin-EDTA (Gibco, Life Technologies) was used to detach cells from the flask surface. They were split in a ratio of 1:5 (ideally 10,000 cells/cm^2^) when they reached 70–80% confluency (every 2–3 days). Transiently transfected Na_V_1.7 HEK293T cells were used in the sodium channel FLIPR assay. HEK293T cells were cultured under 5% carbon dioxide at 37 °C in DMEM Glutamax supplemented with 10% (v/v) FBS. DPBS was used to wash the cells, and 0.25% Trypsin-EDTA was used to detach cells from the flask surface. The cells were split and seeded at 3 million cells per T75 flask, to reach 70–80% confluency after 24 h. The next day, 10 μg plasmid DNA of mouse Na_V_1.7 α subunit (GenScript, Piscataway, USA) was incubated in 500 μL serum-free DMEM Glutamax with 30 μL FuGENE HD transfection reagent (Promega Corporation, Madison, WI, USA) (1:3 DNA/Fugene ratio) for 20 min, and then the mixture was added into the cell flask slowly, drop by drop. After the transfection, the cells were cultured under 5% carbon dioxide at 37 °C for 16 h and then moved to a 28 °C incubator prior to use.

### 4.5. Sodium Channel FLIPR Assay

SH-SY5Y cells or transiently transfected Na_V_1.7 HEK293T cells were seeded into 384-well black wall clear bottom plates at a density of 15,000 cells or 30,000 cells per well, respectively, resulting in 90–95% confluency after 24 h. The media were then removed from the wells and replaced with 20 μL of 10% red membrane potential dye (Molecular Devices, Sunnyvale, CA) in physiological salt solution (PSS) containing 5.9 mM KCl, 1.4 mM MgCl_2_, 10 mM HEPES, 1.2 mM NaH_2_ PO_4_, 5 mM NaHCO_3_, 140 mM NaCl, 11.5 mM glucose, 1.8 mM CaCl_2_ and 0.1% BSA at pH 7.4. The cells were incubated for 30 min at 37 °C in the presence of 5% carbon dioxide. The plates were placed in the FLIPR^TETRA^ (Molecular Devices, Sunnyvale, CA, USA) programmed to record the fluorescence responses under baseline fluorescence 1500–2000 arbitrary fluorescence units (AFU), emission wavelength 565–625 nm, and excitation wavelength 510–545 nm. Prior to the addition of PSS (0.1% BSA) with or without peptide or 10 µM/1 µM TTX, five baseline fluorescence readings were recorded. The fluorescence readings were then recorded once every two seconds over a period of 600 s, resulting in a total of 305 reads before the agonist was added. One fluorescence reading was taken before the second addition. After PSS (0.1% BSA) for negative control or agonist containing 40–50 µM veratridine was loaded, the fluorescence readings were recorded every two seconds for 600 s, resulting in a total 301 reads. *n* independent experiments were conducted in triplicates. Raw fluorescence readings in the form of relative light units were converted to response over baseline using ScreenWorks® (Molecular Devices, version 3.2.0.14) software.

### 4.6. T-type Calcium Channel Window Current FLIPR Assays

HEK293 cells stably expressing Ca_V_3.2 were seeded into 384-well black wall clear bottom plates (Corning, Lowell, MA, US) at a density of 30,000 cells per well. Once the cells reached 80–90% confluency after 24 h, the media were removed from the wells and replaced with 20 μL of 10% calcium 4 dye (Molecular Devices, Sunnyvale, CA, USA) in Hank’s balanced salt solution-HEPES (HBSS-HEPES) (containing 5 mM KCl, 10 mM HEPES, 140 mM NaCl, 10 mM glucose and 0.5 mM CaCl_2_, pH 7.4) with 0.1% bovine serum albumin (BSA). The cells were incubated for 30 min at 37 °C in the presence of 5% carbon dioxide. The plates were placed in the FLIPR^TETRA^ programmed to measure maximum fluorescence intensity following a second addition of the agonist 5 mM CaCl_2_. The data acquisition parameters were adjusted as follows: baseline fluorescence 1500–2000 AFU, emission wavelength 515–575 nm, excitation wavelength 470–495 nm. Prior to the addition of HBSS-HEPES (0.1% BSA) with or without peptide, five baseline fluorescence readings were recorded. The fluorescence readings were then recorded once every two seconds over a period of 600 seconds, resulting in a total of 305 reads before the agonist was added. One fluorescence reading was taken before the second addition. After CaCl_2_ was loaded, the fluorescence readings were recorded every second for 300 s, resulting in a total 301 reads. Raw fluorescence readings in the form of relative light units were converted to response over baseline using ScreenWorks® (Molecular Devices, version 3.2.0.14) software [15].

### 4.7. Whole-Cell Patch-Clamp Electrophysiology

Whole-cell patch-clamp experiments were performed on an automated electrophysiology platform QPatch 16 X (Sophion Bioscience A/S, Ballerup, Denmark) in single-hole configuration using 16-channel planar patch chip QPlates (Sophion Bioscience A/S). The extracellular recording solution contained: 157 mM TEACl, 0.5 mM MgCl_2_, 5 mM CaCl_2_ and 10 mM HEPES; pH 7.4 adjusted with TEAOH; and osmolarity 320 mOsm. The intracellular pipette solution contained: 140 mM CsF, 1 mM EGTA, 10 mM HEPES and 10 mM NaCl; pH 7.2 adjusted with CsOH; and osmolarity 325 mOsm. TxVIA was diluted in extracellular recording solution with 0.1% BSA at the concentrations stated, and the TxVIA effects were compared to the control (extracellular solution with 0.1% BSA) parameters within the same cell. TxVIA incubation time varied from two (for the highest concentration) to five (for the lowest concentration) minutes by applying the voltage protocol 10–30 times at 10 s intervals to ensure steady-state inhibition was achieved. The effects of TxVIA were obtained using 200 ms voltage steps to peak potential from a holding potential of −90 mV. Current–voltage (*I*–*V*) relationships were obtained by holding the cells at a potential of –100 mV before applying 50 ms pulses to potentials from –75 to +50 mV every 5 s in 5 mV increments. Data were fitted with a single Boltzmann distribution: *I*/*I*_max_ = (1 + exp(*V* − *V*_50_)/*k*)^−1^, where *V*_50_ is the half-availability voltage and *k* is the slope factor. A single-voltage protocol was applied in between to measure the TxVIA effect. One cell was considered as an independent experiment. Off-line data analysis was performed using QPatch Assay Software v5.6 (Sophion Bioscience A/S) and Excel 2013 (Microsoft Corporation, Redmond, WA, USA).

### 4.8. Homology Modeling and Molecular Docking

Homology models of human Ca_V_3.2 and Ca_V_3.3 were generated by SWISS-MODEL [39] using human Ca_V_3.1 Cryo-EM structure [24] as template. FASTA sequences for human Ca_V_3.2 and Ca_V_3.3 were obtained from UniProt and used as query sequences. The resulting models were energy minimised using the GROMOS force field, validated by Ramachandran plot analysis, visualised in PyMol and used for molecular docking using Autodock Vina [40]. For molecular docking of TxVIA in human Na_V_1.7, we used the DIV S3-S4 linker of previously published human Na_V_1.7-β1-β2 Cryo-EM structure (PDB 6J8I) [19], as well as the DII S3-S4 linker (missing in the structure of 6J8I) of human Na_V_1.7 Cryo-EM structure (PDB 6N4I) [23]. To define the search space for the DIV S3-S4 linker of the hNa_V_1.7 structure, a grid box with the following dimensions: center *x* = 96.256, center *y* = 136.521, center *z* = 155.042 was used. To define the search space for the DII S3-S4 linker of hNa_V_1.7 structure, a grid box with the following dimensions: center *x* = 82.85, center *y* = 288.228, center *z* = 213.878, was used. The size of the grid box for all the docking in hNa_V_1.7 was as follows: size *x* = 30, size *y* = 30, size *z* = 30. For molecular docking of hCa_V_3.1, hCa_V_3.2, hCa_V_3.3 DIV S3-S4 linker with TxVIA, a grid box with the following dimensions: center *x* = 166.339, center *y* = 123.395, center *z* = 199.419 was used. The size of the grid box for all the docking in hCa_V_3.x was as follows: size *x* = 25, size *y* = 25, size *z* = 25. The exhaustiveness for the search was set to 8.

### 4.9. Evaluation of Zebrafish Pain Behaviours after Intramuscular Injection of TxVIA

Zebrafish were maintained using standard husbandry procedures, conforming to ethical guidelines of the animal ethics committees at the University of Queensland. Six-month old male zebrafish with similar body size and weight were prepared for the assay. Samples were tested via intramuscular injections (5 μL) using a Hamilton syringe (Sigma-Aldrich no. 20795-U). Different concentrations of TxVIA were injected intramuscularly, and the same volume of saline sterilised water was used as control. Three fish were injected per condition. Injected animals were placed in 500 mL containers and their swimming tracks/behaviours were recorded using a custom Zebrabox revolution (Viewpoint) imaging platform and as previously described [41,42]. The animals were observed using the automatic imaging platform for 15 min in dark condition followed by manual monitoring in laboratory light conditions at 28 °C for up to 24 h post-injections. Bursts of erratic swimming were recorded as an indicator of pain behaviours as previously described [14,43].

### 4.10. Data Analysis

Data were plotted and analysed using GraphPad Prism v8.2.1 (GraphPad Software Inc., San Diego, CA, USA). A four-parameter logistic Hill equation with variable Hill coefficients was fitted to the data for concentration-response curves. Data are means ± SEM of *n* independent experiments. Statistical analysis was performed with a paired Student’s *t*-test with statistical significance at *p* < 0.05.

## Figures and Tables

**Table 1:** TxVIA binding affinity in human Na_V_1.7 and Ca_V_3.x.

Docking Target	Molar Affinity (kcal/mol)
hNa_V_1.7 DIV S3-S4	−3.2
hNa_V_1.7 DII S3-S4	7.2
hCa_V_3.1 DIV S3-S4	−4.0
hCa_V_3.2 DIV S3-S4	49.1
hCa_V_3.3 DIV S3-S4	31.8
